# Correction: Fibrinogen and colorectal cancer: A study of the Chinese population

**DOI:** 10.1371/journal.pone.0332402

**Published:** 2025-09-12

**Authors:** Jinfeng Wang, Jing Li, Jing Huo, Yaxi Zhao, Kangkang Liu, Huijie Wang, Xu Cao

The second author’s name is spelled incorrectly. The correct name is: Jing Li.
