# Supplementary material for: Fibrinogen and colorectal cancer: A study of the Chinese population
Source: PLoS One. 2025 May 7;20(5):e0322789. doi: 10.1371/journal.pone.0322789 (PMC12058150; doi:10.1371/journal.pone.0322789)
Supplement: S1 Table — (DOCX) [file pone.0322789.s001.docx]

Table S1 Multivariable logistic regression analyses of fibrinogen and colorectal cancer.

| **Variable** | **Event, n (%)** | **Crude model** | |  | **Model I** | |  | **Model III** | |
| --- | --- | --- | --- | --- | --- | --- | --- | --- | --- |
|  |  | **OR (95% CI)** | ***P*-value** |  | **OR (95% CI)** | ***P*-value** |  | **OR (95% CI)** | ***P*-value** |
| Fibrinogen, g/L | 259/1695 (15.3) | 3.06 (2.55~3.68) | <0.001 |  | 2.54 (2.08~3.1) | <0.001 |  | 1.66 (1.25~2.21) | <0.001 |
| Fibrinogen quartile, g/L | |  |  |  |  |  |  |  |  |
| Q1 (<2.46) | 21/415 (5.1) | 1(ref.) |  |  | 1(ref.) |  |  | 1(ref.) |  |
| Q2 (2.46-2.83) | 26/409 (6.4) | 1.27 (0.7~2.3) | 0.423 |  | 1.34 (0.71~2.54) | 0.369 |  | 1.25 (0.63~2.48) | 0.525 |
| Q3 (2.84-3.30) | 62/446 (13.9) | 3.03 (1.81~5.07) | <0.001 |  | 2.64 (1.5~4.63) | 0.001 |  | 2.36 (1.25~4.43) | 0.008 |
| Q4 (≥3.31) | 150/425 (35.3) | 10.23 (6.32~16.57) | <0.001 |  | 7.7 (4.52~13.12) | <0.001 |  | 3.76 (1.94~7.29) | <0.001 |
| *P* for trend |  |  | <0.001 |  |  | <0.001 |  |  | <0.001 |

Q, quartiles; OR, odds ratio; CI, confidence interval, ALB, albumin; ALT, alanine aminotransferase; AST, aspartate aminotransferase; ALP, alkaline phosphatase; GGT, gamma-glutamyl transferase; ChE, cholinesterase; TBIL, total bilirubin; TBA, total bile acid; GLU, glucose; CREA, creatinine; HDL, high density lipoprotein; LDL, low density lipoprotein; TP, total protein; Lp (a), lipoprotein (a); PLT, platelets; WBC, white blood cell; PT-INR, international normalized ratio of prothrombin time; APTT, activated partial thromboplastin time; TT, thrombin time; CRC, colorectal cancer.

Crude model: no other covariates were adjusted. Model I: adjusted for age and sex. Model II: Model I + weight, family history of CRC, drinking status, smoking status, ALB, ALT, AST, ALP, GGT, ChE, TBIL, TBA, GLU, CREA, urea, HDL, LDL, TP, Lp (a), PLT, WBC, neutrophil count, PT-INR, APTT, TT, hypertension, and liver disease.
