# Supplementary material for: Fibrinogen and colorectal cancer: A study of the Chinese population
Source: PLoS One. 2025 May 7;20(5):e0322789. doi: 10.1371/journal.pone.0322789 (PMC12058150; doi:10.1371/journal.pone.0322789)
Supplement: Attachment — Submitted filename: renamed_9caa2.docx [file pone.0322789.s003.docx]

Dear Editor and reviewers,

On behalf of my co-authors, we greatly appreciate the careful review and comments from both you and the reviewers. We believe that by implementing the suggested changes, we now have a better manuscript entitled “***Fibrinogen and colorectal cancer: A study of Chinese population***” (ID: PONE-D-24-36493) for submission to **PLOS ONE**. We look forward to your positive response to the revised work submitted here.

We present here point-to-point responses for each of the comments in the attached document and have revised our manuscript accordingly. We do not change our raw data or results. Some grammatical and writing errors in the manuscript have been corrected. And we hope the revised manuscript could be acceptable to you. Revised sections are identified with red text in the paper.

There are no conflicts of interest regarding this work. All authors have read the revised manuscript and approved its submission to the **PLOS ONE**. Please do not hesitate to contact us if we can be of any further assistance.

Thank you and best regards.

Yours Sincerely,

Huijie Wang

E-mail: wang_huijie@outlook.com

Shijiazhuang Traditional Chinese Medicine Hospital, NO. 233 Zhongshan West Road, Shijiazhuang, China

# Responds to the reviewer’s comments:

## Reply to Reviewer #1

Thank you very much for your time involved in reviewing the manuscript and your very encouraging comments on the merits.

#### **Comments:**

“This manuscript investigates the relationship between fibrinogen levels and colorectal cancer (CRC) risk specifically within the Chinese population. Analyzing data from 3,595 individuals, including 468 CRC cases and 3,127 controls, collected between April 2015 and June 2022, the researchers utilized logistic regression models and smooth curve fitting for assessment. The findings indicate a significant linear association between elevated fibrinogen levels and an increased risk of CRC, with an adjusted odds ratio of 1.83 [1.52–2.20], P < 0.001. Subgroup and sensitivity analyses further support these conclusions, suggesting that this relationship is stable across various demographic and clinical characteristics. The manuscript is clear and logical, but there are some issues that need improvement. I only recommend the manuscript for publication if the authors can carefully revise the following detailed comments in the manuscript:

1. The rationale behind choosing specific statistical models (e.g., logistic regression) over others should be explained more clearly.

2. Have the findings been externally validated using an independent cohort or dataset?

3. Despite adjusting for multiple confounders, the potential impact of unmeasured factors like dietary habits and physical activity cannot be ignored. These variables could significantly influence both fibrinogen levels and CRC risk. The authors should acknowledge this limitation and discuss its implications for their findings.

4. The choice of statistical models (e.g., logistic regression) over others is not adequately justified. The rationale behind selecting specific methods and cut-off points for quartiles must be better explained, along with their clinical relevance.

5. What specific clinical applications or changes in practice could arise from these findings? How do the authors envision their results impacting patient care or public health strategies?”

##### **Comment 1:**

**“**The rationale behind choosing specific statistical models (e.g., logistic regression) over others should be explained more clearly.”

**Response:**

Thank you for your valuable comments on our manuscript. We would like to provide the following explanations for choosing the logistic regression model:

1. Alignment with Research Objectives:

Case-control studies aim to explore the association between exposure and disease occurrence. Logistic regression can directly estimate the probability relationship between exposure and disease occurrence, obtaining odds ratios (ORs) that clearly demonstrate the impact of exposure factors on disease For instance, in studies examining the relationship between smoking and lung cancer, logistic regression can intuitively determine how much smoking increases the probability of developing lung cancer.

Unlike linear regression, which is used to predict continuous variables, logistic regression is more suitable for binary outcomes in case-control studies.

2. Ability to Handle Multiple Factors:

Disease occurrence in case-control studies is influenced by various factors. Logistic regression can easily incorporate multiple independent variables, such as age, gender, and genetic factors, allowing for simultaneous analysis of their relationship with the disease and adjustment for confounders, accurately assessing the independent effects of each factor.

Compared to models that consider only single factors, logistic regression can comprehensively analyze the combined effects of multiple factors, aligning with real-world scenarios.

3. Flexible Data Distribution Requirements:

Logistic regression does not have strict requirements for data distribution and is applicable to various data types as long as the relationship between the independent variable and the dependent variable meets the logistic relationship assumption. This is advantageous in case-control study data that often do not meet normal distribution requirements.

4. Intuitive Interpretation of Results:

The regression coefficients in logistic regression have clear practical significance; their exponentiated form represents odds ratios (ORs), which intuitively reflect the change in the odds of disease occurrence for each unit change in the independent variable, making it easier for researchers and clinicians to understand and interpret the results.

Many other studies in case-control research have also opted for logistic regression as the statistical method. For instance, certain studies examining the relationship between smoking and lung cancer have utilized logistic regression for data analysis [1-5].

##### **Comment 2:**

“2. Have the findings been externally validated using an independent cohort or dataset?”

**Response:**

Thank you for your insightful question regarding the external validation of our findings. Unfortunately, we did not conduct an external validation using an independent cohort or dataset. We have acknowledged the limitations of our study and made the following updates to clarify these points in the limitation section: “For greater reliability and wider applicability, it is crucial that future research validates these results with independent cohorts.”.

Page 11; Line 224-225.

##### Comment 3:

**“**3. Despite adjusting for multiple confounders, the potential impact of unmeasured factors like dietary habits and physical activity cannot be ignored. These variables could significantly influence both fibrinogen levels and CRC risk. The authors should acknowledge this limitation and discuss its implications for their findings.**”**

**Response:**

Thanks for your great suggestion on improving the accessibility of our manuscript.

Thank you for your valuable feedback. We have revised the relevant section accordingly. The original sentence, "Thirdly, despite controlling for various factors, the potential for unmeasured variables could not be completely ruled out," has been changed to "Thirdly, despite controlling for various factors, unmeasured lifestyle factors (e.g., diet, physical activity), infections, and socioeconomic variables could still influence fibrinogen levels and CRC risk"

Page 10; Line 219-221.

##### Comment 4:

**“**4. The choice of statistical models (e.g., logistic regression) over others is not adequately justified. The rationale behind selecting specific methods and cut-off points for quartiles must be better explained, along with their clinical relevance.**”**

**Response:**

Thank you for your feedback. In case-control studies, logistic regression models are often preferred for several reasons:

1. Alignment with Research Objectives:

Case-control studies aim to explore the association between exposure and disease occurrence. Logistic regression can directly estimate the probability relationship between exposure and disease occurrence, obtaining odds ratios (ORs) that clearly demonstrate the impact of exposure factors on disease For instance, in studies examining the relationship between smoking and lung cancer, logistic regression can intuitively determine how much smoking increases the probability of developing lung cancer.

Unlike linear regression, which is used to predict continuous variables, logistic regression is more suitable for binary outcomes in case-control studies.

2. Ability to Handle Multiple Factors:

Disease occurrence in case-control studies is influenced by various factors. Logistic regression can easily incorporate multiple independent variables, such as age, gender, and genetic factors, allowing for simultaneous analysis of their relationship with the disease and adjustment for confounders, accurately assessing the independent effects of each factor.

Compared to models that consider only single factors, logistic regression can comprehensively analyze the combined effects of multiple factors, aligning with real-world scenarios.

3. Flexible Data Distribution Requirements:

Logistic regression does not have strict requirements for data distribution and is applicable to various data types as long as the relationship between the independent variable and the dependent variable meets the logistic relationship assumption. This is advantageous in case-control study data that often do not meet normal distribution requirements.

4. Intuitive Interpretation of Results:

The regression coefficients in logistic regression have clear practical significance; their exponentiated form represents odds ratios (ORs), which intuitively reflect the change in the odds of disease occurrence for each unit change in the independent variable, making it easier for researchers and clinicians to understand and interpret the results.

Many other studies in case-control research have also opted for logistic regression as the statistical method. For instance, certain studies examining the relationship between smoking and lung cancer have utilized logistic regression for data analysis [1-5].

Additionally, the primary purpose of quartile analysis was to enhance the comprehensibility of the data and to support the results. However, the core findings of the manuscript are based on the restricted cubic spline analysis and the identified threshold effect.

We also performed tertile and quintile analyses using multivariate logistic regression, and the results were similar to those obtained from quartile analysis. These additional analyses further support the robustness and reliability of our findings. The results are as follows:

| Variable | Total, n | Event, n (%) | Crude model | |  | Model I^a^ | |  | Model II^b^ | |
| --- | --- | --- | --- | --- | --- | --- | --- | --- | --- | --- |
|  |  |  | OR (95% CI) | *P*-value |  | OR (95% CI) | *P*-value |  | OR (95% CI) | *P*-value |
| **Tertiles** |  |  |  |  |  |  |  |  |  |  |
| T1 | 1191 | 36 (3) | 1(Ref) |  |  | 1(Ref) |  |  | 1(Ref) |  |
| T2 | 1202 | 106 (8.8) | 3.1 (2.11~4.57) | <0.001 |  | 2.83 (1.88~4.25) | <0.001 |  | 2.93 (1.89~4.52) | <0.001 |
| T3 | 1202 | 326 (27.1) | 11.94 (8.37~17.03) | <0.001 |  | 8.51 (5.84~12.41) | <0.001 |  | 6.53 (4.22~10.11) | <0.001 |
| *P* for trend | 3595 | 468 (13) | 3.59 (3.06~4.2) | <0.001 |  | 2.95 (2.49~3.48) | <0.001 |  |  | <0.001 |
| Quintiles |  |  |  |  |  |  |  |  |  |  |
| Q1 | 717 | 13 (1.8) | 1(Ref) |  |  | 1(Ref) |  |  | 1(Ref) |  |
| Q2 | 710 | 36 (5.1) | 2.89 (1.52~5.5) | 0.001 |  | 2.58 (1.33~5.03) | 0.005 |  | 2.53 (1.27~5.04) | 0.008 |
| Q3 | 705 | 59 (8.4) | 4.95 (2.69~9.1) | <0.001 |  | 4.24 (2.25~7.98) | <0.001 |  | 4.33 (2.23~8.42) | <0.001 |
| Q4 | 741 | 112 (15.1) | 9.64 (5.38~17.29) | <0.001 |  | 7.29 (3.97~13.4) | <0.001 |  | 7.51 (3.91~14.43) | <0.001 |
| Q5 | 722 | 248 (34.3) | 28.33 (16.03~50.08) | <0.001 |  | 19.22 (10.62~34.8) | <0.001 |  | 13.75 (7.11~26.59) | <0.001 |
| *P* for trend | 3595 | 468 (13) |  | <0.001 |  |  | <0.001 |  |  | <0.001 |

OR, odds ratio; CI, confidence interval; Q, quartile; ALB, albumin; GGT, gamma-glutamyl transferase; ALP, alkaline phosphatase; AST, aspartate aminotransferase; ALT, alanine aminotransferase; ChE, cholinesterase; TBA, total bile acid; TBIL, total bilirubin; HDL, high density lipoprotein; LDL, low density lipoprotein; GLU, glucose; CREA, creatinine; TP, total protein; Lp(a), lipoprotein(a); PLT, platelets; WBC, white blood cell; PT-INR, international normalized ratio of prothrombin time; TT, thrombin time; APTT, activated partial thromboplastin time; CRC, colorectal cancer.

^a^Adjusted for age and sex. ^b^Adjusted for Model I + weight, family history of CRC, drinking status, smoking status, ALB, ALT, AST, ALP, GGT, ChE, TBIL, TBA, GLU, CREA, urea, HDL, LDL, TP, Lp(a), PLT, WBC, neutrophil count, PT-INR, APTT, TT, hypertension, and liver disease.

##### Comment 5:

**“**5. What specific clinical applications or changes in practice could arise from these findings? How do the authors envision their results impacting patient care or public health strategies?**”**

**Response:**

Thank you for reviewing our work and for your valuable feedback. We have added this section to the discussion section of the manuscript:

“We discovered a non-linear, inverse L-shaped relationship between fibrinogen levels and CRC risk, pinpointing a critical threshold at 3.794 g/L. This finding provides significant evidence for targeting fibrinogen levels as a means of reducing CRC risk through clinical interventions. Notably, when fibrinogen levels are controlled below 3.794 g/L, CRC risk decreases substantially as fibrinogen levels drop. The threshold offers vital evidence for managing fibrinogen levels. Nevertheless, the potential diagnostic utility of fibrinogen can be enhanced when used in conjunction with other tumor markers such as carcinoembryonic antigen and prealbumin, possibly leading to improved diagnostic performance in CRC [6]. Future clinical trials and studies are needed to validate the effectiveness of fibrinogen as a diagnostic marker and to further explore its role in the adenoma-carcinoma sequence.”

Page 10; Line 206-215.

## Reply to Reviewer #2

Thank you again for your positive comments and valuable suggestions to improve the quality of our manuscript. And we hope the revised manuscript could be acceptable for you.

#### Comments:

“This manuscript provides valuable insights into the association between fibrinogen and CRC in the Chinese population. However, several aspects require further elaboration to enhance the robustness and interpretability of the findings.

1. The manuscript sometimes implies a causal relationship between fibrinogen and CRC (e.g., "fibrinogen promotes cancer progression"). However, as this is a cross-sectional and retrospective study, causality cannot be inferred. Reframe the language throughout the manuscript to emphasize association rather than causality.

2. Although the study adjusts for numerous confounders (e.g., BMI, smoking, liver disease), certain lifestyle factors (diet, exercise), acute or chronic infective conditions, and socioeconomic variables are missing. These may influence both fibrinogen levels and CRC risk.

3. The study highlights a linear association between fibrinogen and CRC risk. However, non-linear relationships or threshold effects are not thoroughly investigated. Consider performing and reporting restricted cubic spline analyses to assess potential non-linear associations. This could reveal critical fibrinogen thresholds that significantly elevate CRC risk. Furthermore, if non-linear patterns are observed, discuss how clinical guidelines might use fibrinogen cut-off values to stratify CRC risk in patients.

4. Expand the discussion by integrating more references on the thrombin-fibrinogen axis or tumor microenvironment, and how fibrinogen promotes tumor growth, angiogenesis, and metastasis in CRC patients.

5. Abbreviations like ALP, AST, WBC, etc. used earlier in the text should be consistently applied in the subsequent sensitivity analysis section.

6. The link between fibrinogen and colorectal cancer might be attributed to chronic inflammation and the changes in the body's internal environment caused by tumors, such as coagulation issues. This is similar to other inflammatory markers like C-reactive protein, which also lack specificity. The studies mentioned by the author in the discussion section only show a connection between fibrinogen and colorectal cancer, but they don't establish that high levels of fibrinogen are a risk factor for the disease. Therefore, the author should provide a clearer explanation of the significance of their research. It's widely understood that any malignant tumor can lead to chronic inflammation and is associated with related inflammatory markers.”

##### Comment 1:

“1. The manuscript sometimes implies a causal relationship between fibrinogen and CRC (e.g., "fibrinogen promotes cancer progression"). However, as this is a cross-sectional and retrospective study, causality cannot be inferred. Reframe the language throughout the manuscript to emphasize association rather than causality.”

**Response 1:**

Thank you for your insightful comments. We acknowledge your concern regarding the potential implication of a causal relationship between fibrinogen and colorectal cancer (CRC). We agree that, given the nature of our study, causality cannot be inferred. We have revised the manuscript to reframe the language, emphasizing association rather than causality throughout the text. Below are some key changes we have made to address your feedback:

Revised statements to consistently use terms like "association" and "relationship" instead of terms that imply causation.

Added clarifications in the discussion to highlight that our findings indicate associations rather than causative relationships: “Although this retrospective observational study cannot confirm a causal relationship between fibrinogen and CRC”. Page 9; Line 188-189.

The sentence “fibrinogen promotes cancer progression” was deleted.

##### Comment 2:

“2. Although the study adjusts for numerous confounders (e.g., BMI, smoking, liver disease), certain lifestyle factors (diet, exercise), acute or chronic infective conditions, and socioeconomic variables are missing. These may influence both fibrinogen levels and CRC risk.”

**Response 2:**

Thanks for your great suggestion on improving the accessibility of our manuscript.

Thank you for your valuable feedback. We have revised the relevant section accordingly. The original sentence, "Thirdly, despite controlling for various factors, the potential for unmeasured variables could not be completely ruled out," has been changed to "Thirdly, despite controlling for various factors, unmeasured lifestyle factors (e.g., diet, physical activity), infections, and socioeconomic variables could still influence fibrinogen levels and CRC risk"

Page 10; Line 219-221.

##### Comment 3:

“3. The study highlights a linear association between fibrinogen and CRC risk. However, non-linear relationships or threshold effects are not thoroughly investigated. Consider performing and reporting restricted cubic spline analyses to assess potential non-linear associations. This could reveal critical fibrinogen thresholds that significantly elevate CRC risk. Furthermore, if non-linear patterns are observed, discuss how clinical guidelines might use fibrinogen cut-off values to stratify CRC risk in patients.”

**Response 3:**

Thank you for your valuable feedback and guidance, particularly regarding the non-linear relationships and threshold effects. We have conducted restricted cubic spline analyses as suggested to evaluate potential non-linear associations. In this re-analysis, we used 99.5% of the data sample, a correction from the previous 99% data sample usage.

Our findings indicate a non-linear relationship between fibrinogen levels and colorectal cancer (CRC) risk. Specifically, as shown in Table 3, when fibrinogen levels are below 3.794 g/L, the CRC risk significantly increases (OR 5.944, 95% CI 4.084–8.65, P <0.001). However, when fibrinogen levels are above 3.794 g/L, the risk stabilizes (OR 0.838, 95% CI 0.431–1.632, P = 0.604).

The Result section amended as specified below:

The restricted cubic spline analysis (Fig 2, 99.5% data) revealed a non-linear relationship between fibrinogen levels and CRC risk, identifying a threshold at 3.794 g/L. Specifically, below this threshold, the CRC risk significantly increases (OR 5.944, 95% CI 4.084–8.65, P <0.001), whereas, above this threshold, the association is no longer statistically significant (OR 0.838, 95% CI 0.431–1.632, P = 0.604).

**Fig 2. Association between fibrinogen and colorectal cancer.** Adjusted for age, sex, weight, family history of colorectal cancer, drinking status, smoking status, albumin, gamma-glutamyl transferase, alanine aminotransferase, alkaline phosphatase, aspartate aminotransferase, cholinesterase, total bilirubin, total bile acid, glucose, creatinine, urea, total protein, low-density lipoprotein, high-density lipoprotein, lipoprotein(a), platelets, white blood cell, neutrophil count, the international normalized ratio of prothrombin time, activated partial thromboplastin time, thrombin time, hypertension, and liver disease. Only 99.5% of the data is shown.

**Table 3. Threshold effect analysis of the relationship of fibrinogen and CRC.**

| Fibrinogen, g/L | Adjusted Model | |
| --- | --- | --- |
|  | OR (95% CI) | P-value |
| <3.794 | 5.944 (4.084~8.65) | <0.001 |
| ≥3.794 | 0.838 (0.431~1.632) | 0.604 |
| Likelihood Ratio test | | <0.001 |

OR, odds ratio; CI, confidence interval; ALB, albumin; GGT, gamma-glutamyl transferase; ALP, alkaline phosphatase; AST, aspartate aminotransferase; ALT, alanine aminotransferase; ChE, cholinesterase; TBA, total bile acid; TBIL, total bilirubin; HDL, high density lipoprotein; LDL, low density lipoprotein; GLU, glucose; CREA, creatinine; TP, total protein; Lp(a), lipoprotein(a); PLT, platelets; WBC, white blood cell; PT-INR, international normalized ratio of prothrombin time; TT, thrombin time; APTT, activated partial thromboplastin time; CRC, colorectal cancer.

Adjusted for age and sex, weight, family history of CRC, drinking status, smoking status, ALB, ALT, AST, ALP, GGT, ChE, TBIL, TBA, GLU, CREA, urea, HDL, LDL, TP, Lp(a), PLT, WBC, neutrophil count, PT-INR, APTT, TT, hypertension, and liver disease. Page 7-8; Line 129-150.

Additionally, we have discussed how clinical guidelines might use fibrinogen cut-off values to stratify CRC risk in patients. These findings suggest that the threshold effect of fibrinogen levels can serve as a valuable clinical indicator for CRC risk assessment and prediction. While we have confirmed this non-linear relationship and its threshold effect, further studies are needed to validate these results and explore the underlying biological mechanisms.

Discussion section:

“We discovered a non-linear, inverse L-shaped relationship between fibrinogen levels and CRC risk, pinpointing a critical threshold at 3.794 g/L. This finding provides significant evidence for targeting fibrinogen levels as a means of reducing CRC risk through clinical interventions. Notably, when fibrinogen levels are controlled below 3.794 g/L, CRC risk decreases substantially as fibrinogen levels drop. The threshold offers vital evidence for managing fibrinogen levels. Additionally, the potential diagnostic utility of fibrinogen can be enhanced when used in conjunction with other tumor markers such as carcinoembryonic antigen and prealbumin, possibly leading to improved diagnostic performance in CRC [6]. Future clinical trials and studies are needed to validate the effectiveness of fibrinogen as a diagnostic marker and to further explore its role in the adenoma-carcinoma sequence.” Page 10; Line 206-215.

Other corresponding parts have also been corrected.

Abstract section:

“Logistic regression models and restricted cubic spline analysis were employed to assess the association between fibrinogen and CRC. When fibrinogen levels were divided into quartiles, the ORs for CRC in Q2, Q3, and Q4 compared to Q1 were 2.72 (95% CI: 1.57–4.74), 4.84 (95% CI: 2.84–8.25), and 9.55 (95% CI: 5.55–16.43), respectively. Restricted cubic spline analysis identified a non-linear association between fibrinogen levels and CRC risk with a threshold of 3.794 g/L. Below this threshold, the CRC risk significantly increased (OR 5.944, 95% CI 4.084–8.65, P < 0.001). These findings may offer new insights for the diagnosis and management of CRC.” Page 2; Line 26-32.

Discussion section:

“In this case-control study, we found that fibrinogen levels are positively correlated with CRC risk. Subgroup analyses further confirmed the stability of this association. Notably, we identified an inverse L-shaped relationship between fibrinogen levels and CRC risk, with a significant threshold at 3.794 g/L.” Page 8-9; Line 170-173.

Conclusion section:

“Fibrinogen levels are positively correlated with CRC risk, and the relationship between them exhibits an inverse L-shape, with a critical turning point at 3.794 g/L. Future clinical trials and studies are necessary to further validate the role of fibrinogen in CRC clinical management.” Page11; Line 227-229.

##### Comment 4:

4. Expand the discussion by integrating more references on the thrombin-fibrinogen axis or tumor microenvironment, and how fibrinogen promotes tumor growth, angiogenesis, and metastasis in CRC patients.

**Response 4:**

Thank you for your valuable feedback. To address your suggestion, we have expanded the discussion section by integrating more references on the thrombin-fibrinogen axis and the tumor microenvironment, as well as the role of fibrinogen in promoting tumor growth, angiogenesis, and metastasis in CRC patients.

We amended the following paragraph:

“Although this retrospective observational study cannot confirm a causal relationship between fibrinogen and CRC, evidence from both basic and clinical research indicates an interaction between cancer and the hemostatic system. Cancer may promote thromboembolism, while pathological activation of the hemostatic system can drive cancer progression. The thrombin-fibrinogen axis plays a crucial role in CRC [7]. Fibrinogen interacts with leukocyte integrin αMβ2, promoting inflammatory responses that lead to increased stress and phosphorylation of proliferation markers in intestinal epithelial cells, indicating a direct link between fibrinogen/inflammatory function and tumorigenic changes in colon epithelial cells [8]. Fibrinogen promotes angiogenesis by stimulating endothelial cell migration, proliferation, and tubular structure formation, and it binds growth factors such as bFGF and VEGF, protecting them from degradation and enhancing endothelial cell activation, thereby reinforcing angiogenesis [9]. Additionally, the fibrin matrix plays a key role in platelet stabilization and activation, and platelets promote the expansion and function of immunosuppressive myeloid cells during the early stages of inflammation-induced adenoma formation, and their infiltration into colon adenomas, further driving tumor cell metastasis [10]. These mechanisms collectively promote tumor growth, angiogenesis, and metastasis in CRC patients through the interplay of the inflammatory and coagulation axes. Conversely, tumors can induce hypoxia and hypercoagulable states in vivo [11, 12], leading to elevated fibrinogen levels in CRC patients, which are a result of tissue damage and subsequent inflammatory responses [13].” Page 9-10; Line 188-205.

##### Comment 5:

“5. Abbreviations like ALP, AST, WBC, etc. used earlier in the text should be consistently applied in the subsequent sensitivity analysis section.”

**Response 5:**

Thank you for your feedback. We have reviewed the sensitivity analysis section and figure captions. The full terms mentioned are specifically in the caption for Figure 2. Using full terms in figure captions ensures precise expression and clarity for the readers. Please find the caption for Figure 2 below:

Fig 2. Association between fibrinogen and colorectal cancer. Except for the subgroup component itself, each subgroup factor was adjusted for age, sex, weight, family history of colorectal cancer, drinking status, smoking status, albumin, gamma-glutamyl transferase, alanine aminotransferase, alkaline phosphatase, aspartate aminotransferase, cholinesterase, total bilirubin, total bile acid, glucose, creatinine, urea, total protein, low-density lipoprotein, high-density lipoprotein, lipoprotein(a), platelets, white blood cell, neutrophil count, the international normalized ratio of prothrombin time, activated partial thromboplastin time, thrombin time, hypertension, and liver disease.

Page 8; Line 155-162.

##### Comment 6:

“6. The link between fibrinogen and colorectal cancer might be attributed to chronic inflammation and the changes in the body's internal environment caused by tumors, such as coagulation issues. This is similar to other inflammatory markers like C-reactive protein, which also lack specificity. The studies mentioned by the author in the discussion section only show a connection between fibrinogen and colorectal cancer, but they don't establish that high levels of fibrinogen are a risk factor for the disease. Therefore, the author should provide a clearer explanation of the significance of their research. It's widely understood that any malignant tumor can lead to chronic inflammation and is associated with related inflammatory markers.”

**Response 6:**

Thank you for your valuable comments. I would like to address a few key points to clarify and deepen the understanding of our study:

1) Link between Fibrinogen and CRC: As you pointed out, elevated fibrinogen levels may result from chronic inflammation and environmental changes induced by tumors. This phenomenon is not unique to CRC; it applies to various malignancies. However, our study confirmed the consistency and stability of our results by using a large sample of data and through various analyses.

The corresponding paragraph referred to was amended.

“Our findings are in line with previous investigations exploring the relationship between fibrinogen and CRC in the general population. These studies have consistently suggested that elevated levels of fibrinogen are independently and positively associated with CRC risk [14, 15]. For example, a prospective study involving 84,000 individuals from the general population in Denmark found that high levels of fibrinogen were associated with an increased risk of CRC. Similarly, research from the Moli-Sani cohort indicated that individuals. These studies suggest that fibrinogen may be an important biomarker for assessing CRC risk, potentially offering new approaches and methods for the early diagnosis and treatment of CRC.” Page 9; Line 174-181.

2) Correlation versus Causation: We fully recognize that our current study is a retrospective case-control design, which primarily reveals correlation rather than causation. Nevertheless, future prospective cohort studies and clinical trials are expected to validate and further elucidate the potential causal role of fibrinogen in the pathogenesis of CRC. We have reflected this acknowledgment in the limitations section. “Furthermore, our study design, being retrospective, primarily reveals correlation rather than causation.” Page 10-11; Line 221-222.

3) Clinical Implications: Our findings suggest that individuals with relatively high levels of fibrinogen may benefit from more vigilant monitoring and early screening to detect potential tumors. Fibrinogen, as a non-specific but critically important marker of inflammation, in combination with other tumor markers such as carcinoembryonic antigen and prealbumin, may provide more comprehensive diagnostic information and improve CRC screening performance. We have added this key point to the Discussion section. “We discovered a non-linear, inverse L-shaped relationship between fibrinogen levels and CRC risk, pinpointing a critical threshold at 3.794 g/L. This finding provides significant evidence for targeting fibrinogen levels as a means of reducing CRC risk through clinical interventions. Notably, when fibrinogen levels are controlled below 3.794 g/L, CRC risk decreases substantially as fibrinogen levels drop. The threshold offers vital evidence for managing fibrinogen levels. Nevertheless, the potential diagnostic utility of fibrinogen can be enhanced when used in conjunction with other tumor markers such as carcinoembryonic antigen and prealbumin, possibly leading to improved diagnostic performance in CRC [6]. Future clinical trials and studies are needed to validate the effectiveness of fibrinogen as a diagnostic marker and to further explore its role in the adenoma-carcinoma sequence.” Page 10; Line 206-215.

### Reference

1.	Shaib YH, El-Serag HB, Davila JA, Morgan R, McGlynn KA. Risk factors of intrahepatic cholangiocarcinoma in the United States: A case-control study. Gastroenterology. 2005;128(3):620-6. doi: 10.1053/j.gastro.2004.12.048.

2.	Phillips MR, Yang G, Zhang Y, Wang L, Ji H, Zhou M. Risk factors for suicide in China: a national case-control psychological autopsy study. The Lancet. 2002;360(9347):1728-36. doi: 10.1016/S0140-6736(02)11681-3.

3.	Velayos FS, Loftus EV, Jess T, Harmsen WS, Bida J, Zinsmeister AR, et al. Predictive and Protective Factors Associated With Colorectal Cancer in Ulcerative Colitis: A Case-Control Study. Gastroenterology. 2006;130(7):1941-9. doi: 10.1053/j.gastro.2006.03.028.

4.	Davila JA, Morgan RO, Shaib Y, McGlynn KA, El-Serag HB. Diabetes increases the risk of hepatocellular carcinoma in the United States: a population based case control study. Gut. 2005;54(4):533. doi: 10.1136/gut.2004.052167.

5.	Hassan MM, Bondy ML, Wolff RA, Abbruzzese JL, Vauthey J-N, Pisters PW, et al. Risk Factors for Pancreatic Cancer: Case-Control Study. Am J Gastroenterol. 2007;102(12).

6.	Ying H-Q, Chen W, Xiong C-F, Wang Y, Li X-J, Cheng X-X. Quantification of fibrinogen-to-pre-albumin ratio provides an integrating parameter for differential diagnosis and risk stratification of early-stage colorectal cancer. Cancer Cell Int. 2022;22(1):137. doi: 10.1186/s12935-022-02532-y.

7.	Cantrell R, Palumbo JS. The thrombin–inflammation axis in cancer progression. Thromb Res. 2020;191:S117-S22. doi: 10.1016/S0049-3848(20)30408-4.

8.	Steinbrecher KA, Horowitz NA, Blevins EA, Barney KA, Shaw MA, Harmel-Laws E, et al. Colitis-Associated Cancer Is Dependent on the Interplay between the Hemostatic and Inflammatory Systems and Supported by Integrin αMβ2 Engagement of Fibrinogen. Cancer Res. 2010;70(7):2634-43. doi: 10.1158/0008-5472.CAN-09-3465.

9.	Staton CA, Brown NJ, Lewis CE. The role of fibrinogen and related fragments in tumour angiogenesis and metastasis. Expert Opin Biol Ther. 2003;3(7):1105-20. doi: 10.1517/14712598.3.7.1105.

10.	Servais L, Wéra O, Dibato Epoh J, Delierneux C, Bouznad N, Rahmouni S, et al. Platelets contribute to the initiation of colitis-associated cancer by promoting immunosuppression. J Thromb Haemostasis. 2018;16(4):762-77. Epub 2018/01/26. doi: 10.1111/jth.13959. PubMed PMID: 29369476.

11.	Lawrence SO, Simpson-Haidaris PJ. Regulated de novo biosynthesis of fibrinogen in extrahepatic epithelial cells in response to inflammation. Thromb Haemost. 2004;92(2):234-43. Epub 2004/07/23. doi: 10.1160/th04-01-0024. PubMed PMID: 15269818.

12.	Sahni A, Simpson-Haidaris PJ, Sahni SK, Vaday GG, Francis CW. Fibrinogen synthesized by cancer cells augments the proliferative effect of fibroblast growth factor-2 (FGF-2). J Thromb Haemostasis. 2008;6(1):176-83. Epub 2007/10/24. doi: 10.1111/j.1538-7836.2007.02808.x. PubMed PMID: 17949478.

13.	Beer JH, Haeberli A, Vogt A, Woodtli K, Henkel E, Furrer T, et al. Coagulation markers predict survival in cancer patients. Thromb Haemost. 2002;88(5):745-9. Epub 2002/11/13. PubMed PMID: 12428088.

14.	Parisi R, Panzera T, Russo L, Gamba S, De Curtis A, Di Castelnuovo A, et al. Fibrinogen levels in relation to colorectal cancer onset: A nested case-cohort study from the Moli-sani cohort. Front Cardiovasc Med. 2022;9. doi: 10.3389/fcvm.2022.1009926.

15.	Allin KH, Bojesen SE, Nordestgaard BG. Inflammatory biomarkers and risk of cancer in 84,000 individuals from the general population. Int J Cancer. 2016;139(7):1493-500. doi: 10.1002/ijc.30194.
