# Supplementary material for: Fibrinogen and colorectal cancer: A study of the Chinese population
Source: PLoS One. 2025 May 7;20(5):e0322789. doi: 10.1371/journal.pone.0322789 (PMC12058150; doi:10.1371/journal.pone.0322789)
Supplement: Attachment — Submitted filename: renamed_8927d.docx [file pone.0322789.s004.docx]

Dear Editor and reviewers,

On behalf of my co-authors, we greatly appreciate the careful review and comments from both you and the reviewers. We believe that by implementing the suggested changes, we now have a better manuscript entitled “***Fibrinogen and colorectal cancer: A study of Chinese population***” (ID: PONE-D-24-36493) for submission to **PLOS ONE**. We look forward to your positive response to the revised work submitted here.

We present here point-to-point responses for each of the comments in the attached document and have revised our manuscript accordingly. We do not change our raw data or results. Some grammatical and writing errors in the manuscript have been corrected. And we hope the revised manuscript could be acceptable to you. Revised sections are identified with red text in the paper.

There are no conflicts of interest regarding this work. All authors have read the revised manuscript and approved its submission to the **PLOS ONE**. Please do not hesitate to contact us if we can be of any further assistance.

Thank you and best regards.

Yours Sincerely,

Huijie Wang

E-mail: wang_huijie@outlook.com

Shijiazhuang Traditional Chinese Medicine Hospital, NO. 233 Zhongshan West Road, Shijiazhuang, China

# Responds to the reviewer #1’s comments:

## Reply to Reviewer #1

Thank you very much for your time involved in reviewing the manuscript and your very encouraging comments on the merits.

#### **Comments:**

“The revisions have strengthened the manuscript, particularly in clarifying methodological choices, acknowledging limitations, and expanding the discussion. Below are my remaining observations and suggestions for further improvement:

1. The authors provided a detailed rationale for using logistic regression, including alignment with study objectives, handling of confounders, and intuitive interpretation of ORs. Tertile analyses further support the robustness of quartile-based findings.

2. The study involves multiple subgroup analyses and sensitivity analyses, which increase the risk of Type I errors (false positives). The authors do not mention any adjustments for multiple testing.

3. The authors should provide more concrete recommendations for clinical practice, such as specific scenarios where fibrinogen monitoring could be beneficial.

4. The sensitivity analysis with complete cases (n=1,695) shows consistent results, but the manuscript should report the proportion of missing data and imputation methods in the Methods section.

5. Ensure the restricted cubic spline plot clearly labels the threshold (3.794 g/L) and includes confidence intervals.”

##### **Comment 1:**

“1. The authors provided a detailed rationale for using logistic regression, including alignment with study objectives, handling of confounders, and intuitive interpretation of ORs. Tertile analyses further support the robustness of quartile-based findings.”

**Response:**

Thank you for your positive feedback. We appreciate your recognition of our work.

##### **Comment 2:**

“2. The study involves multiple subgroup analyses and sensitivity analyses, which increase the risk of Type I errors (false positives). The authors do not mention any adjustments for multiple testing.”

**Response:**

Thank you for your valuable feedback. We appreciate your suggestion regarding the need for multiple testing correction.

We would like to clarify that the interaction P-values for all subgroups (e.g., sex, age, HLP, hypertension, ischemic cerebrovascular disease, and CHD) were greater than 0.05, indicating no statistically significant differences in the relationship between fibrinogen and CRC across these subgroups. As such, we believe that the association is consistent across different subgroups, and there is no evidence of significant effect modification. In light of these findings, even if a Bonferroni correction were applied, the results would remain unchanged, as none of the interaction P-values would reach the adjusted significance threshold.

##### Comment 3:

“3. The authors should provide more concrete recommendations for clinical practice, such as specific scenarios where fibrinogen monitoring could be beneficial.”

**Response:**

Thanks for your great suggestion on improving the accessibility of our manuscript. We have supplemented the portion of the discussion that deals with this aspect. This paragraph is specified below:

We discovered a non-linear, inverse L-shaped relationship between fibrinogen levels and CRC risk, identifying a critical threshold at 3.794 g/L. This finding provides strong evidence supporting the use of fibrinogen levels in clinical interventions aimed at reducing CRC risk. Notably, when fibrinogen levels are controlled below 3.794 g/L, CRC risk decreases significantly as fibrinogen levels decline, highlighting the importance of this threshold in managing fibrinogen levels. Additionally, the diagnostic utility of fibrinogen may be enhanced when combined with other tumor markers, such as carcinoembryonic antigen and prealbumin, which could potentially improve CRC diagnostic performance [44]. Future clinical trials and studies are needed to further explore the role of fibrinogen in the adenoma-carcinoma sequence and validate its effectiveness as a diagnostic biomarker. Upon such validation, monitoring fibrinogen levels could become a valuable risk assessment tool prior to colonoscopy in CRC screening, owing to its efficiency, speed, and non-invasive nature. When elevated fibrinogen levels are detected, they should be assessed in conjunction with other risk factors to evaluate CRC risk, and colonoscopy should be employed for screening high-risk individuals.

Page 10; Line 208-220.

##### Comment 4:

“4. The sensitivity analysis with complete cases (n=1,695) shows consistent results, but the manuscript should report the proportion of missing data and imputation methods in the Methods section.”

**Response:**

Thank you for your valuable comments. Based on your suggestion, we have modified the following sentence in the Methods section to clarify the methodology used in the sensitivity analysis:

The sentence has been changed from:

“Furthermore, this study used multiple imputations (five replications) and sensitivity analysis with complete cases.”

To:

“Missing data were handled using multiple imputations by chained equations with five replications. In addition, a sensitivity analysis using complete cases was performed to assess the robustness of the results.”

Page 5; Line 93-95.

##### Comment 5:

“5. Ensure the restricted cubic spline plot clearly labels the threshold (3.794 g/L) and includes confidence intervals.”

**Response:**

Thank you for your valuable feedback. In response to your suggestion, we have updated the restricted cubic spline plot to clearly label the threshold, now set at 3.794 g/L, with the associated confidence intervals (95% CI: 3.747–3.841).

Additionally, we have made corresponding updates in both the **Results** and **Abstract** sections to reflect these changes.
